# Glucocorticoid Receptor Expression Predicts Good Outcome in response to Taxane-Free, Anthracycline-Based Therapy in Triple Negative Breast Cancer

**DOI:** 10.1155/2020/3712825

**Published:** 2020-05-20

**Authors:** Ahmed Elkashif, Victoria Bingham, Paula Haddock, Matthew P. Humphries, Stephen McQuaid, Paul B. Mullan, Helen O. McCarthy, Niamh E. Buckley

**Affiliations:** ^1^School of Pharmacy, Queen's University Belfast, Belfast BT9 7BL, UK; ^2^Patrick G Johnston Centre for Cancer Research, Queen's University Belfast, Belfast BT9 7AE, UK

## Abstract

Triple negative breast cancer (TNBC) is a poor outcome subset of breast cancers characterised by the lack of expression of ER *α*, PR, and HER2 amplification. It is a heterogeneous group of cancers which fail to derive benefit from modern, more targeted treatments such as Tamoxifen and Herceptin. Current standard of care (SoC) is cytotoxic chemotherapy, which is effective for some patients, with other patients deriving little/no benefit and lacking alternative treatments. This study has identified the glucocorticoid receptor (GR) as a potential predictive biomarker of response to anthracycline-based chemotherapy in triple negative breast cancer (TNBC). GR gene expression levels in patient samples were analysed through publicly available microarray datasets as well as protein expression through immunohistochemistry (IHC) and correlated with clinical/pathological outcomes, including survival. While the results confirmed previous observations that high GR expression is associated with poor outcome in response to taxane-based chemotherapy, this study shows for the first time that high GR expression is associated with improved outcomes in the context of anthracycline-based chemotherapy. GR therefore has the potential to be used as a predictive biomarker to guide treatment choices and ensure that patients derive the greatest benefit from first line treatment, avoiding unnecessary costs, side effects, and disease progression.

## 1. Introduction

Breast cancer is the most common malignancy in females worldwide, with over 2 million new cases diagnosed in 2018 and an estimated 600,000 deaths [1]. Following diagnosis of breast cancer, patients are stratified based on expression of estrogen receptor alpha (ER*α*), progesterone receptor (PR), and amplification of the human epidermal growth factor receptor 2 (HER2) gene. This is used to guide treatments, with targeted treatments including Tamoxifen and Trastuzumab (Herceptin) used to treat ER*α* positive and HER2 positive cancers, respectively.

Triple negative breast cancer (TNBC) is a term used to describe breast cancers which are ER*α*/PR negative and lack amplification of the HER2 gene. This subset is associated with the poorest outcomes among breast cancers, with higher mortality rates compared to other subtypes, despite accounting for only 15–20% of cases [2]. This is due, in part, to the lack of molecular targets available for treatment. Given the lack of biomarkers to guide treatment, first line therapy for TNBC is a regimen of adjuvant and increasingly neoadjuvant, cytotoxic chemotherapy consisting of anthracyclines and taxanes. Despite the high rates of mortality, TNBC has higher response rates to chemotherapy compared to other breast cancers. This is commonly referred to as the “TNBC paradox”. In the neoadjuvant setting, patients who achieve a complete pathological response (pCR) tend to have a very good prognosis and survival rates comparable to non-TNBC, accounting for around a third of patients [2]. Those who do not show a response to first line chemotherapy or have residual disease (RD) following treatment tend to relapse in the first three years following diagnosis and have a high risk of disease progression and death [2, 3]. This highlights the importance of biomarkers to stratify patients and tailor treatment options accordingly and that an optimal response to first line treatment is paramount in assuring long term survival. Given the fact that TNBC is a diagnosis of exclusion (based on the lack if receptors, as opposed to the presence), this variable response to therapy is not surprising. Without the use of additional biomarkers, such diagnostic criteria leave a highly heterogeneous population with variable treatment response rates that in theory should be able to be further stratified. This is exemplified by the further subclassification of TNBC into the Vanderbilt subtypes by Lehmann et al. consisting of four subgroups, each with distinctive gene expression patterns, responses to chemotherapy, and overall outcomes [4, 5].

The development of personalised medicine approaches in TNBC is an area of increasing interest with the identification and development of novel targeted treatments and/or combinations as well as the associated companion biomarkers [6]. A major advancement in this field has been the development of immunotherapy and the use of immune checkpoint inhibitors. TNBC is characterised by a high expression of immune checkpoint proteins such as PD-L1 as well as a degree of tumour infiltrating lymphocytes (TILs), both of which are predictive of response to immunotherapy as well as conventional chemotherapies such as anthracyclines [6–9]. Clinical trials have shown that response rates to pembrolizumab, a monoclonal antibody targeting PD-1, are increased from 5% to 18.5% by assessing PD-L1 status and only treating patients expressing this marker [10, 11]. Additionally, the KEYNOTE-355 phase III clinical trial has shown the addition of pembrolizumab to chemotherapy improves outcome in metastatic TNBC with the primary end point of progression-free survival being met (NCT02819518). Other targets being explored in TNBC include the androgen receptor (AR), epidermal growth factor receptor (EGFR), vascular endothelial growth factor (VEGF), and cyclin dependent kinases (CDKs). The preclinical and clinical development of these areas has been reviewed by ourselves and others; however, their incorporation into routine clinical practice to date has been limited [6, 12, 13]. With research into these novel targets ongoing, the current reliance is still on cytotoxic chemotherapy, primarily anthracycline-based. These treatments have an important role, with many patients deriving benefit from their use and achieving long-term survival. We sought to identify a biomarker for SoC chemotherapy in TNBC to differentiate between good and poor responders which allows chemotherapy to be used effectively and alternative treatments to be offered where necessary.

In this study, we show that high expression of GR is indicative of patients who will respond well to anthracycline-based chemotherapy regimens. GR is a steroid hormone receptor expressed ubiquitously in the body and is responsible for a range of functions, regulating inflammation, response to stress, and cell survival. It exists in the cell cytoplasm, bound to heat shock proteins (HSPs). Its endogenous ligand is cortisol and upon ligand binding, GR dissociates from HSPs, dimerises, and translocates to the nucleus. It binds to glucocorticoid response elements (GREs) on DNA to transactivate or transrepress genes. GR is involved in transrepression of genes such as those responsible for inflammation (e.g., AP1 and NF*κ*B) while transactivating other genes such as those responsible for cell survival and DNA repair (e.g., STAT1 and Ets) [14–16].

This study implicates GR as a potential prognostic biomarker in the context of SoC which could be used clinically to guide treatment choices and tailor chemotherapy to individual patients.

## 2. Materials and Methods

### 2.1. Gene Expression

The in-house gene expression dataset has been previously described [17]. The publicly available datasets GSE5881 [18], GSE31519 [19], GSE7390 [20], GSE10797 [21], and GSE9574 [22] were accessed online using NCBI.

### 2.2. Tissue Microarray (TMA) and Immunohistochemistry (IHC)

The TMAs described in this study were constructed from formalin fixed paraffin embedded (FFPE) slides prepared by the Northern Ireland Biobank (NIB13-0043) and the Breast Cancer Now Tissue Bank (TR-0055). Both the NIB (REC:11/NI/0013) and the Breast Cancer Now Tissue Bank have ethical approval to use deidentified tissue samples from NHS tissue pathology archives (specifically the Belfast Health and Social Care Trust Cellular Pathology archive in the case of the NIB). The first TMA with matched samples to the in-house gene expression cohort has been previously described elsewhere [17, 23]. The subsequent TMAs with various chemotherapy regimens have also been previously described [9, 24–26]. Clinical information from these TMAs is summarised in Table 1. IHC - was performed in the Northern Ireland Molecular Pathology Laboratory, which has UK Clinical Pathology Accreditation. Tumour sections were cut from TMA blocks for H&E staining to check for quality. Following this, 4 *µ*m sections were cut, dried overnight at 37°C, and stained using an automated immunostainer (Leica Bond-Max, Milton Keynes, UK). TNBC status was confirmed as previously described [27]. Epitope retrieval solution was applied to the sections for 20 minutes followed by a GR specific monoclonal antibody (Cell Signalling, #3660) used at a dilution of 1 : 50 for 15 minutes. Sections were visualised with diaminobenzidine, counterstained with haematoxylin for 5 minutes, and then mounted on a Sakura Autostainer.

### 2.3. Assessment

All IHC cores were assessed by at least two experienced immunohistochemists blinded to clinical and pathological data. For the IHC analysis, an H score approach to grading the level of GR expression was adopted. This was obtained by assigning a cell intensity score for each core on a scale of 0–3. This number was multiplied by the relative percentage of positive cells, giving a range of scores from 0–300. Cores with significant fragmentation or cores with no identifiable tumour cells were excluded from analysis. Data from replicate cores were averaged to represent the case. A summary of assigned scores for each TMA can be found in Supplementary Table1.

### 2.4. Survival Analysis and Statistics

Kaplan–Meier analysis and hazard ratio calculations were carried out using GraphPad Prism (v8.2.1). Remaining data were analysed using two-tailed *t*-tests or one-way ANOVA tests as appropriate. Data were deemed significant with a *P* value of < 0.05 signified by^*∗*^ and < 0.01 by^*∗∗*^.

## 3. Results

**Figure 1:**
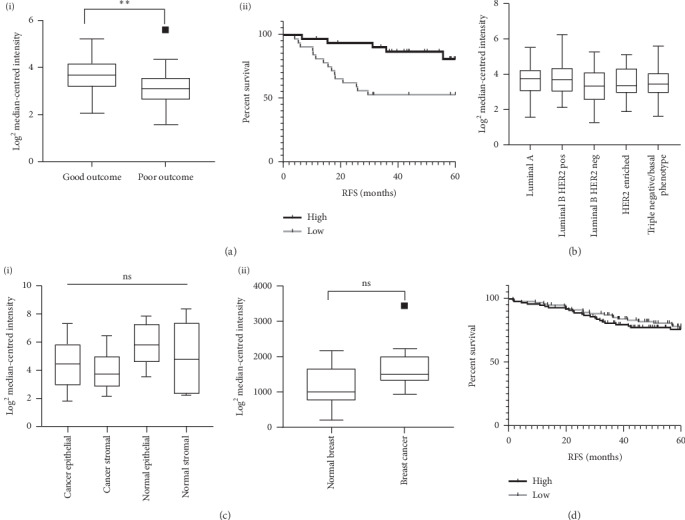
(a) (i) Box and whisker plot showing GR expression in good (RFS > 3 years) and poor (RFS < 3 years) outcome patients from the in-house TNBC dataset. (ii) Kaplan–Meier curve of relapse free survival in the in-house TNBC dataset based on GR gene expression above (high) and below (low) the median. (b) Box and whisker plot showing GR expression in each of the St. Gallen subtypes in the in-house dataset. (c) Box and whisker plots showing GR expression in normal versus cancerous breast tissue in the publicly available datasets: (i) GSE10797 and (ii) GSE9574. 1 (d) Kaplan–Meier curve of relapse free survival of the entire in-house cohort dichotomised based on GR expression above (high) and below (low) the median.

Preceding the present study, an *in silico* gene expression analysis was carried out to identify genes associated with good or poor outcome in TNBC [23]. Differentially expressed genes were identified in an in-house cohort of 30 good outcome (no relapse within 3 years) and 30 poor outcome (relapse within 3 years) patients treated with FEC (fluorouracil, epirubicin, cyclophosphamide) based chemotherapy. One of the genes most significantly associated with good outcome was NR3C1, encoding GR (*P*=0.0028 (Figure 1(a) (i)). Furthermore, expression of GR was significantly associated with improved relapse free survival (HR 0.27 (CI 0.1118–0.6519) *P*=0.0064) (Figure 1(a) (ii)).

We next expanded the analysis to a cohort which consisted of 205 FEC treated patients spanning the molecular subgroups of breast cancer as defined by the St. Gallen classifications. These are IHC based subgroups of breast cancer based on ER*α*, PR, HER2 and Ki67% expression and are used as prognostic guides, to accurately predict disease features and survival [28]. No significant difference in GR expression was observed between any of the St. Gallen groups (Figure 1(b)). While no differences in GR expression within breast cancer was observed, we next wanted to investigate whether GR expression varied between normal tissue and breast cancer. Using two publicly available datasets (GSE10797 and GSE9574) [21, 22], there were no significant differences in GR expression between normal and cancerous tissue, including epithelial and stromal cells (Figure 1(c)).

We went on to investigate whether the observed association between GR expression and improved outcome was specific to TNBC. High GR expression was only associated with improved survival in the TNBC subset of patients, with no significant difference observed in survival when compared to all other subsets either combined (Figure 1(d)) or individually (Supplementary Figure 1(a)).

**Figure 2:**
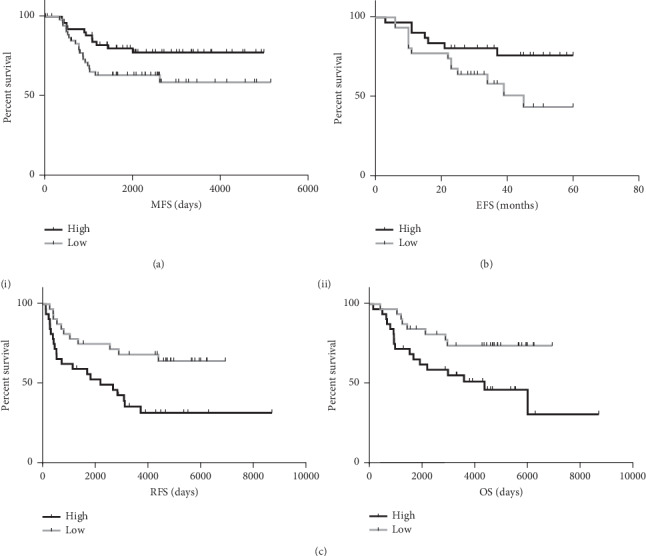
Kaplan–Meier curves of (a) metastasis and (b) event-free survival dichotomised based on GR expression above (high) or below (low) the median in the publicly available datasets GSE58812 (*N* = 107) and GSE31519 (*N* = 62), respectively. (c) Kaplan–Meier curves of (i) relapse free survival and (ii) overall survival dichotomised based on GR expression above (high) or below (low) the median in the publicly available dataset GSE7390 (*N* = 64).

To validate these findings, we identified two independent publicly available TNBC datasets with clinical follow-up and available gene expression information (GSE58812 and GSE31519) [18, 19]. High GR expression was significantly associated with improved metastasis free survival (MFS) (HR 0.4843 (CI 0.2392-0.9805) *P*=0.0478) (Figure 2(a)) and improved event free survival (EFS) (HR 0.4108 (CI 0.1739–0.9703) *P*=0.0432) (Figure 2(b)).

To verify whether GR was prognostic of TNBC pathology and disease progression or whether it was a predictive marker of response to chemotherapy, we identified an untreated cohort of ER negative patients from the TRANSBIG study (GSE7390) [20]. In this cohort, high GR expression was associated with significantly poorer relapse free survival (RFS) (HR 2.553 (CI 1.267–5.142) *P*=0.0087) and overall survival (OS) (HR 2.615 (CI 1.189–5.751) *P*=0.0196) (Figure 2(c)). This indicates that high GR expression is indicative of worse overall disease progression but predicts patients who may respond best to FEC based chemotherapy.

While gene expression provides valuable information, IHC examining protein expression fits more readily within the routine diagnostic pipeline. We therefore interrogated GR protein expression through IHC analysis on tissue microarrays (TMAs) stained for GR in tumour samples.

**Figure 3:**
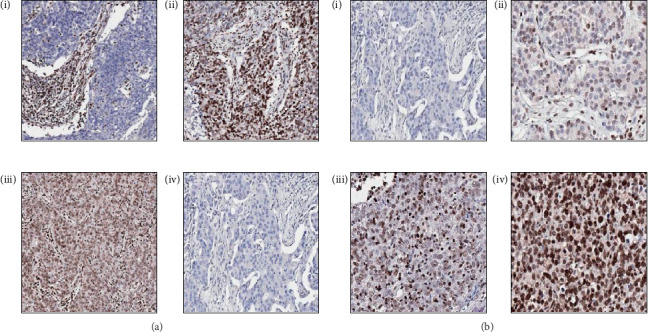
(a) Representative images showing staining patterns of GR in breast cancer tissue, including (i) low tumour, high stroma; (ii) high tumour, low stroma; (iii) high tumour, high stroma; and (iv) low tumour low stroma. (b) Representative images showing scoring strategy including H scores of (i) 0, (ii) 100, (iii) 200, and (iv) 300. All TMA images taken at x20 magnification.

In the breast cancer TMAs, a range of expression was observed in both the epithelial and stromal compartments (Figure 3(a)). At the cellular level, GR was localised to the nucleus, consistent with its role as a nuclear receptor and transcription factor. As expression varied by intensity and percentage of cells with expression, an H score was used to capture GR expression within the epithelial and stromal compartments separately. Following preliminary analysis, only tumour scores were taken further for subsequent analyses as GR expression in stromal cells showed little variation with the vast majority exhibiting strong positive staining. Representative images outlining the scoring strategy are shown in Figure 3(b).

**Figure 4:**
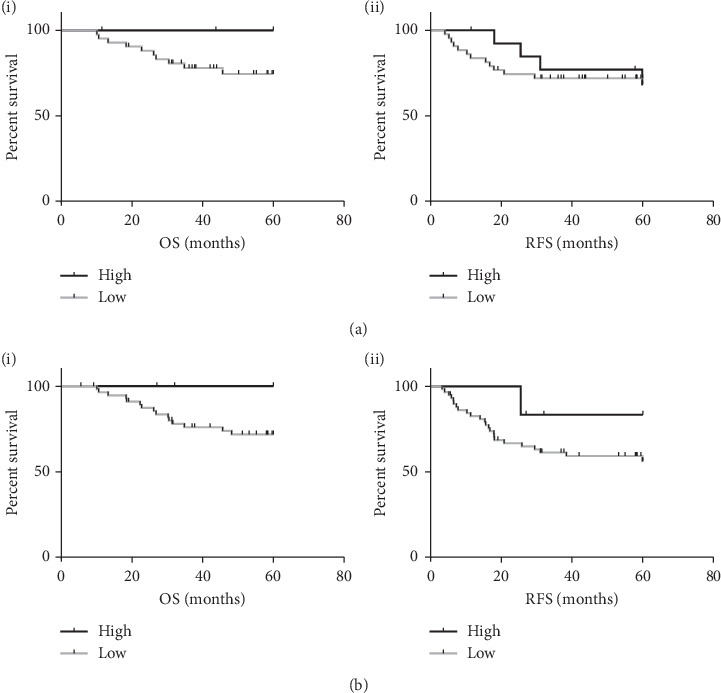
Kaplan–Meier curves of (i) overall survival and (ii) relapse-free survival stratified by high and low GR IHC expression in (a) TMA #1 (*N* = 57) and (b) TMA #2 (*N* = 64).

The influence of GR protein expression on survival was first analysed on a TMA with matched samples to the in-house gene expression analysis (TMA #1). Consistent with previous findings, high expression of GR was associated with significantly improved OS (HR 0.2296 (CI 0.06689–0.7882) *P* = 0.0194) and an improved RFS which failed to reach significance (Figure 4(a)). A second TNBC cohort was identified and scored for GR expression (TMA #2) with similar results derived. High GR protein expression in tumour cells was found to be associated with improved RFS and OS in this cohort (Figure 4(b)). Despite a strong association, this did not reach significance likely due to the low sample number limiting the statistical power of the analysis.

**Figure 5:**
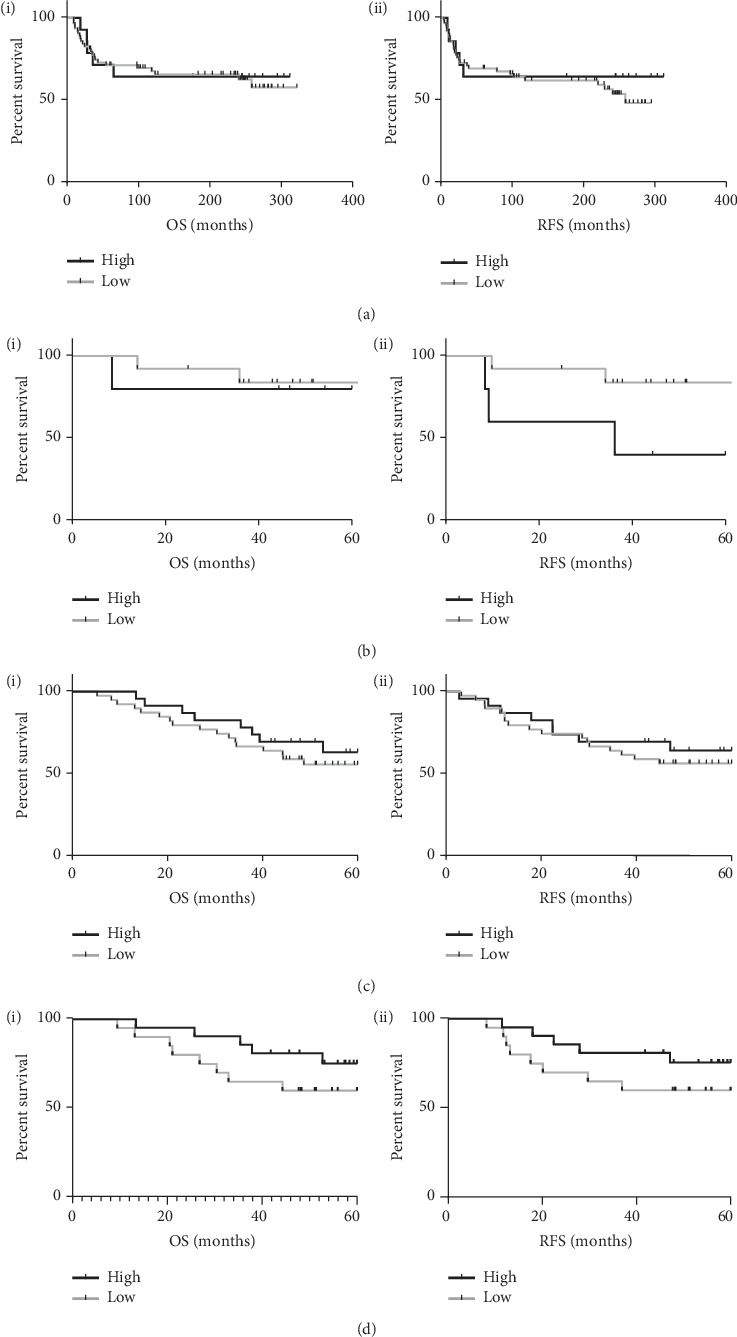
Kaplan–Meier curves of (i) overall survival and (ii) relapse-free survival stratified by GR IHC expression in the cohorts: (a) TMA #3 CMF treated (*N* = 77), (b) TMA #3 taxane treated (*N* = 18), (c) TMA #4 all chemo (*N* = 62), and (d) TMA #4 anthracycline treated (*N* = 41).

As our discovery and validation datasets were FEC treated, we next wanted to look at the relationship between GR and outcome in the context of other chemotherapies. We therefore analysed two additional TMAs with a variety of chemotherapies used within the cohorts (TMA #3 and TMA #4, respectively). The first of these had primarily two regimens: one consisting of cyclophosphamide, methotrexate, and 5-fluorouracil (CMF) and a second consisting of 5-fluorouracil, epirubicin, cyclophosphamide, and docetaxel (FEC-D). These were analysed separately to investigate the effect of removing the anthracycline (CMF) and adding the taxane (FEC-D) to therapy. In the CMF treated patients, there was no association between GR expression and survival (RFS and OS) (Figure 5(a)). However, in the patients with a taxane (docetaxel) added to FEC chemotherapy, high GR expression was associated with decreased RFS (Figure 5(b)). This failed to reach significance as there were only 18 patients treated with this regimen, which led to a low statistical power. This finding, however, is interesting given the previous link established between high GR expression and poor survival following taxane treatment [14, 29]. The second TMA used to examine the effect of chemotherapy on the predictive power of GR consisted of 27 FEC and 14 AC (adriamycin, cyclophosphamide) treated patients out of a total of 56 patients with treatments, also including FEC-D or no chemotherapy. In the total cohort, there is no association between GR expression and outcome (Figure 5(c)). However, when the analysis is restricted to anthracycline treated patients (FEC/AC), it appears that high GR expression is again associated with improved RFS and OS (Figure 5(d)). Similarly to the previous analyses, this fails to reach significance due to the low patient numbers in this cohort. However, when the data from TMA cohorts #2 and #3 consisting of taxane-free, anthracycline treated patients are combined, high GR expression is significantly associated with improved RFS (HR 0.4466 (CI 0.2225–0.8965), *P*=0.0442) (Supplementary Figure1(b)).

Summaries of statistical analyses carried out above are shown in Supplementary Tables 2–6.

## 4. Discussion

The results of the present study indicate that high gene and/or protein expression of GR is indicative of patients who will respond well to anthracycline-based chemotherapy without the use of taxanes. This appears to be specific to TNBC. Conversely, high GR expression appears to be indicative of patients who will respond poorly to regimens which include taxanes.

GR is of particular interest in the context of oncology due to the widespread use of GR agonists in the treatment of chemotherapy induced nausea and vomiting. Glucocorticoids such as dexamethasone are prescribed alongside chemotherapy for this purpose. Adherence to these medications is mainly symptom led and varies greatly between patients, although it is required before administration of taxane-based treatments [30, 31]. Such agents may, through GR signalling, be affecting response to chemotherapy and therefore outcome in TNBC. This highlights the clinical relevance of studies such as this.

A number of previous studies have made the link between GR signalling and disease progression/outcome/chemotherapy response in TNBC and other cancers. High GR has been associated with decreased overall survival in ovarian cancer [32]. Additionally, it has been proposed that glucocorticoids may promote breast cancer metastasis through upregulation of pathways associated with metastasis such as epithelial mesenchymal transition, glucose metabolism, and epidermal growth factor receptor signalling [33]. The role of GR in TNBC has also previously been interrogated. Pan et al. have found that expression of GR was associated with poor outcome in TNBC [29]. This was proposed to be caused by the inhibition of taxane-based chemotherapy induced apoptosis by GR signalling. GR responsive genes such as serum and glucocorticoid inducible protein kinase-1 (SGK1) and mitogen-activated protein kinase phosphatase-1 (MKP1) were implicated in this process [34]. This relationship appears to contradict our findings. However, this can be explained by the fact that our findings show a relationship between high GR expression and improved survival in the context of anthracycline-based chemotherapy regimens without the use of taxanes. Such regimens are the first line treatment of choice in TNBC. It would appear that the utility of GR as a biomarker is twofold, predicting which patients will respond well to anthracycline-based chemotherapy regimens, as well as those who will respond poorly to taxane-based treatments. We further explored the relationship between GR expression and response to taxanes in a small number of patients fitting these criteria, with the results agreeing with the findings of Pan et al. and Wu et al. [29, 34].

This chemotherapy dependent role of GR can further be inferred from a 2018 study which examined the effect of glucocorticoid use on survival in patients with stages I–III breast cancer. The use of glucocorticoids in patients receiving no systemic chemotherapy was found to be associated with more aggressive clinical features such as higher histological grade and lymph node involvement [35]. Glucocorticoid use was found to be associated with smaller tumours and less lymph node involvement among anthracycline treated patients. Additionally, glucocorticoid use was significantly associated with prolonged OS in ER*α* negative patients and shorter OS in ER*α* positive patients. The results of our study show that hormone receptor status and choice of chemotherapy both influence the role that GR plays as a biomarker and its potential use as a treatment target. These are consistent with our findings that high GR expression predicts good outcome in the context of ER*α* negative/TNBC and anthracycline-based chemotherapy.

There are a number of GR related pathways that could explain how signalling could affect response to chemotherapy, DNA damaging, or otherwise. It has been revealed that glucocorticoids may induce the production of reactive oxygen species (ROS) in breast cancer cells [36]. ROS can cause DNA damage and could have a synergistic effect when combined with DNA damaging chemotherapies such as anthracyclines [37, 38]. Taxanes on the other hand produce low levels of ROS; thus, no synergy would be expected [37].

Another pathway that could be implicated in GR modulating chemotherapy response is the NF*κ*B signalling pathway. GR is a known regulator of NF*κ*B [39], but the literature is conflicting on the nature of this relationship. Two studies have demonstrated the beneficial effects of NF*κ*B signalling on the efficacy of chemotherapy *in vitro* and *in vivo*. One study found that the addition of dexamethasone increased the cytotoxicity of cisplatin in human cervical carcinoma cell line [40]. The other showed increased antitumour activity of Adriamycin (doxorubicin), gemcitabine, and carboplatin against breast cancer cells when combined with dexamethasone in mice [41, 42]. Both studies postulated that this activity was due to GR induced inhibition of NF*κ*B leading to increased apoptosis following chemotherapy. However, data from our research group has suggested that, in the presence of BRCA1 dysfunction, high NF*κ*B signalling plays a role in improving outcome in TNBC by recruiting immune cells such as CD8+ cytotoxic T cells, creating an antitumour microenvironment and preventing progression [17]. As observed from the present study, context in terms of hormone receptor status and chemotherapy is key in deducing the role of such pathways, but the effect of GR on NF*κ*B is potentially a key mechanism behind the predictive effect of this marker.

## 5. Conclusions

In conclusion, we have identified that the expression of GR is predictive of TNBC patients that will respond well to anthracycline-based chemotherapy, which is the current SoC in the UK and other countries. Such markers can be easily incorporated into routine IHC-based testing and can be used to guide effective treatment choices at an early stage. This also has potential implications for the use of glucocorticoids alongside chemotherapy regimens as this could be beneficial with anthracyclines and detrimental with taxanes. The development of such markers is essential in heterogeneous populations such as TNBC to stratify patients into clinically relevant populations in order to adopt a personalised approach to disease treatment. For the successful validation of such biomarkers, large TNBC populations must be analysed for expression of GR and response to chemotherapy, both anthracycline, and non-anthracycline-based. Such studies will carry sufficient statistical power to validate GR as a predictive biomarker.

## Figures and Tables

**Table 1:** Clinical information for the TMAs: (A) TMA #1, (B) TMA #2, (C) TMA #3, and (D) TMA #4.

*(A) TMA #1 clinical information*		
Median age (range)		50 (28–74)
Grade	1 2 3	0 6 51
Chemotherapy		FEC

*(B) TMA #2 clinical information*		
Median age (range)		49 (28–74)
Grade	1 2 3	0 7 57
Chemotherapy		FEC

*(C) TMA #3 clinical information*		
Median age (range)		45 (28–76)
Grade	1 2 3 Not stated	0 6 105 1
Chemotherapy	CMF FEC-docetaxel Tamoxifen/radiotherapy	77 18 17

*(D) TMA #3 clinical information*		
Median age (range)		54 (36–82)
Grade	1 2 3	0 6 56
Chemotherapy	FEC AC TACT-FEC None	27 14 8 13

## Data Availability

The gene expression datasets analysed in the present study are available from the NCBI repository (https://www.ncbi.nlm.nih.gov/gds). All TMA samples are available upon application from the Northern Ireland Biobank (http://www.nibiobank.org/) and the Breast Cancer Now Tissue Bank (https://www.breastcancertissuebank.org/).

## Supplementary Materials

Supplementary Materials Supplementary Figure 1: A,Kaplan Meier Curve of relapse free survival in the in-house dataset based on GR gene expression above (high) and below (low) the median split into the St. Gallen subgroups, (i) Luminal A (*N* = 77), (ii) Luminal B HER2 positive (*N* = 61), (iii) Luminal B HER2 negative (*N* = 30), and (iv) HER2 enriched (*N* = 37). B, Kaplan Meier curve of relapse free survival in anthracycline treated TNBC patients in TMA #2 and TMA #4 combined (*N* = 105). Supplementary Table 1: log rank hazard ratios, 95% confidence intervals, and *P* values for survival analysis of metastasis/event free survival dichotomised based on below (low) or above (high) median gene expression of GR in the publicly available datasets, GSE58812 and GSE31519, respectively. Supplementary Table 2: log rank hazard ratios, 95% confidence intervals, and *P* values for survival analysis of metastasis/event free survival dichotomised based on below (low) or above (high) median gene expression of GR in the publicly available dataset, GSE7390. Supplementary Table 3: log rank hazard ratios, 95% confidence intervals, and *P* values for survival analysis relapse free/overall survival stratified by high or low GR IHC expression in (A) TMA #1 and (B) TMA #2. Supplementary Table 4: log rank hazard ratios, 95% confidence intervals, and *P* values for survival analysis relapse free/overall survival stratified by high or low GR IHC expression in (A) CMF treated patients in TMA #3 and (B) taxane treated patients in TMA #3. Supplementary Table 5: log rank hazard ratios, 95% confidence intervals, and *P* values for survival analysis relapse free/overall survival stratified by high or low GR IHC expression in (A) all patients in TMA #4 and (B) anthracycline treated patients in TMA #4.
