# Supplementary material for: Glucocorticoid Receptor Expression Predicts Good Outcome in response to Taxane-Free, Anthracycline-Based Therapy in Triple Negative Breast Cancer
Source: J Oncol. 2020 May 20;2020:3712825. doi: 10.1155/2020/3712825 (PMC7256765; doi:10.1155/2020/3712825)
Supplement: Supplementary Materials — Supplementary Figure 1: A,Kaplan Meier Curve of relapse free survival in the in-house dataset based on GR gene expression above (high) and below (low) the median split into the St. Gallen subgroups, (i) Luminal A (N = 77), (ii) Luminal B HER2 positive (N = 61), (iii) Luminal B HER2 negative (N = 30), and (iv) HER2 enriched (N = 37). B, Kaplan Meier curve of relapse free survival in anthracycline treated TNBC patients in TMA #2 and TMA #4 combined (N = 105). Supplementary Table 1: log rank hazard ratios, 95% confidence intervals, and P values for survival analysis of metastasis/event free survival dichotomised based on below (low) or above (high) median gene expression of GR in the publicly available datasets, GSE58812 and GSE31519, respectively. Supplementary Table 2: log rank hazard ratios, 95% confidence intervals, and P values for survival analysis of metastasis/event free survival dichotomised based on below (low) or above (high) median gene expression of GR in the publicly available dataset, GSE7390. Supplementary Table 3: log rank hazard ratios, 95% confidence intervals, and P values for survival analysis relapse free/overall survival stratified by high or low GR IHC expression in (A) TMA #1 and (B) TMA #2. Supplementary Table 4: log rank hazard ratios, 95% confidence intervals, and P values for survival analysis relapse free/overall survival stratified by high or low GR IHC expression in (A) CMF treated patients in TMA #3 and (B) taxane treated patients in TMA #3. Supplementary Table 5: log rank hazard ratios, 95% confidence intervals, and P values for survival analysis relapse free/overall survival stratified by high or low GR IHC expression in (A) all patients in TMA #4 and (B) anthracycline treated patients in TMA #4. [file 3712825.f1.pdf]

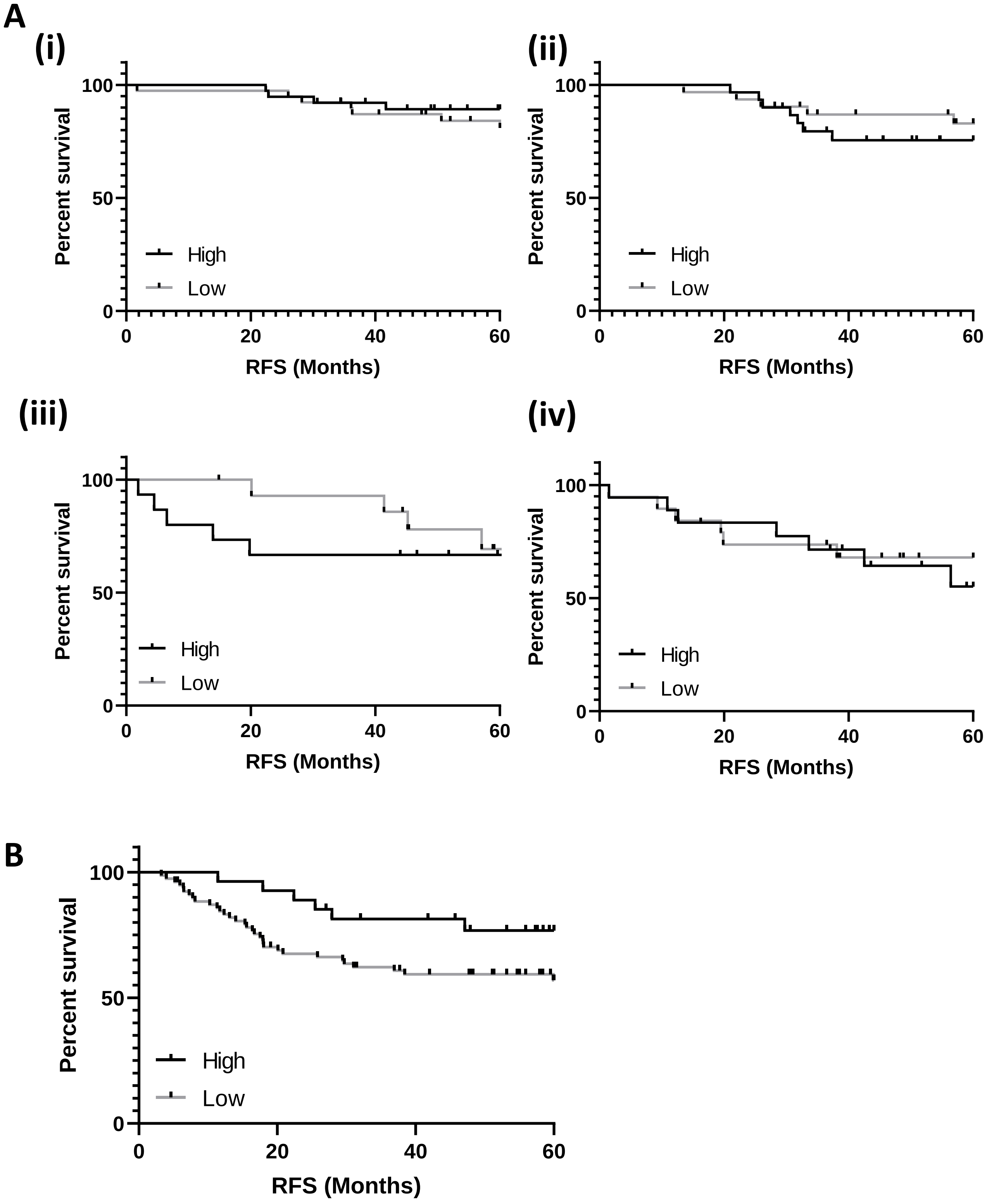

Supp Figure 1 A Kaplan Meier Curve of relapse free survival in the in-house dataset based on GR gene expression above (high) and below (low) the median split into the St Gallen subgroups (i) Luminal A (N=77), (ii) Luminal B HER2 positive (N=61), (iii) Luminal B HER2 negative (N=30) (iv) HER2 Enriched (N=37).

B Kaplan Meier curve of relapse free survival in anthracycline treated TNBC patients in TMA #2 and TMA #4 combined (N=105).

Supp Table 1 Summary of assigned IHC H-scores in the tumour and stromal compartments in TMAs #1-4.

TMA #1 (N=57)	Tumour Score	Cases	Stroma Score	Cases
	0	14	0	3
	1-100	32	1-100	10
	101-200	6	101-200	21
	201-300	5	201-300	23
**TMA #2 (N=64)**	**Tumour Score**	**Cases**	**Stroma Score**	**Cases**
	0	16	0	6
	1-100	30	1-100	10
	101-200	12	101-200	15
	201-300	6	201-300	33
**TMA #3 (N=112)**	**Tumour Score**	**Cases**	**Stroma Score**	**Cases**
	0	19	0	3
	1-100	43	1-100	7
	101-200	35	101-200	37
	201-300	15	201-300	65
**TMA #4 (N=62)**	**Tumour Score**	**Cases**	**Stroma Score**	**Cases**
	0	26	0	6
	1-100	13	1-100	6
	101-200	12	101-200	19
	201-300	11	201-300	31

Supp. Table 2 Log rank hazard ratios, 95% confidence intervals and p-values for survival analysis of metastasis/event free survival dichotomised based on below (low) or above (high) median gene expression of GR in the publicly available datasets, GSE58812 and GSE31519, respectively

		N (n)	HR	95% CI	P-value
**Metastasis Free Survival**		107 (31)			
**GR**	Low	54 (20)	1		
	High	53 (11)	0.4843	0.2392-0.9805	0.0478*
**Event Free Survival**		62 (21)			
**GR**	Low	31 (14)	1		
	High	31 (7)	0.4108	0.1739-0.9703	0.0432*

Supp. Table 3 Log rank hazard ratios, 95% confidence intervals and p-values for survival analysis of metastasis/event free survival dichotomised based on below (low) or above (high) median gene expression of GR in the publicly available dataset, GSE7390.

GSE 7390		N (n)	HR	95% CI	P-value
**Relapse Free Survival**		64 (32)			
**GR**	Low	32 (11)	1		
	High	32 (21)	2.553	1.267-5.142	0.0087**
**Overall Survival**		64 (32)			
**GR**	Low	32 (8)	1		
	High	32 (17)	2.615	1.189-5.751	0.0196*

Supp. Table 4 Log rank hazard ratios, 95% confidence intervals and p-values for survival analysis relapse free/overall survival stratified by high or low GR IHC expression in (A) TMA #1 and (B) TMA #2.

**A**<br>**TMA #1**		**N (n)**	**HR**	**95% CI**	**P-value**
**Relapse Free Survival**		57 (16)			
**GR**	Low	43 (12)	1		
	High	14 (4)	0.8724	0.2917-2.609	0.8122
**Overall Survival**		57 (12)			
**GR**	Low	42 (12)	1		
	High	15 (0)	0.2296	0.06689-0.7882	0.0194*

**B**<br>**TMA #2**		**N (n)**	**HR**	**95% CI**	**P-value**
**Relapse Free Survival**		64 (26)			
**GR**	Low	58 (25)	1		
	High	6 (1)	0.3189	0.09401-1.082	0.2365
**Overall Survival**		63 (18)			
**GR**	Low	57 (18)	1		
	High	6 (0)	0.3201	0.07484-1.370	0.1246

Supp. Table 5 Log rank hazard ratios, 95% confidence intervals and p-values for survival analysis relapse free/overall survival stratified by high or low GR IHC expression in (A) CMF treated patients in TMA #3 and (B) Taxane treated patients in TMA #3.

**A**<br>**TMA #3 CMF**		**N (n)**	**HR**	**95% CI**	**P-value**
**Relapse Free Survival**		77 (32)			
**GR**	Low	63 (27)	1		
	High	14 (5)	0.7407	0.3115-1.761	0.5343
**Overall Survival**		77 (28)			
**GR**	Low	63 (23)	1		
	High	14 (5)	0.9010	0.3534-2.298	0.8324

**B**<br>**TMA #3 Taxane**		**N (n)**	**HR**	**95% CI**	**P-value**
**Relapse Free Survival**		18 (5)			
**GR**	Low	13 (2)	1		
	High	5 (3)	4.939	0.6210-39.28	0.2365
**Overall Survival**		18 (3)			
**GR**	Low	13 (2)	1		
	High	5 (1)	1.424	0.1079-18.79	0.7717

Supp. Table 6 Log rank hazard ratios, 95% confidence intervals and p-values for survival analysis relapse free/overall survival stratified by high or low GR IHC expression in (A) All patients in TMA #4 and (B) Anthracycline treated patients in TMA #4.

**A**<br>**TMA #4 All**		**N (n)**	**HR**	**95% CI**	**P-value**
**Relapse Free Survival**		62 (25)			
**GR**	Low	39 (17)	1		
	High	23 (15)	0.7726	0.3443-1.734	0.5458
**Overall Survival**		62 (25)			
**GR**	Low	39 (17)	1		
	High	23 (15)	0.7301	0.3271-1.630	0.4610

**B**<br>**TMA #4 Anthracycline**		**N (n)**	**HR**	**95% CI**	**P-value**
**Relapse Free Survival**		41 (13)			
**GR**	Low	21 (5)	1		
	High	20 (8)	0.5175	0.1736-1.542	0.2390
**Overall Survival**		41 (13)			
**GR**	Low	21 (5)	1		
	High	20 (8)	0.4929	0.1649-1.473	0.2038
